# Effect of implant angulation and impression technique on Nobel active implant impressions: An original study

**DOI:** 10.6026/973206300211191

**Published:** 2025-05-31

**Authors:** Prasanthi G.S, Uppala Sushma, Tikkisetty Chaitanya Jyothi, Varchaswi Vellanki, Raguri Manaswi, Neha Agrawal

**Affiliations:** 1 Department of Prosthodontics and Implantology, Sibar Institute of Dental Sciences, Takkelapadu, Guntur andhra Pradesh, India; 2 Department of Conservative and Endodontics, Sibar Institute of Dental Sciences, Takkelapadu, Guntur andhra Pradesh, India; 3 Department of Biochemistry, Index Medical College Hospital and Research Center, Indore, India

**Keywords:** Nobel active implants, impression techniques, open-tray, closed-tray, implant angulation, impression accuracy, coordinate measuring machine, prosthetic fit, implant-supported restoration

## Abstract

Accurate implant impressions are essential for the success and longevity of implant-supported prostheses. The Nobel Active system has a tapered body and aggressive thread design, demanding high precision. Therefore, it is of interest to compare open-tray and closed-tray techniques for impression accuracy. Implants were placed at 0°, 15°, and 25° angulations for evaluation. A Coordinate Measuring Machine was used for 3D deviation measurements. At 0°, both techniques showed negligible differences in accuracy. At 15° and 25°, the open-tray technique had significantly better accuracy (p < 0.05). Impression errors increased with implant angulation, mainly in closed-tray groups. Angulated implants posed greater challenges for accurate impressions. Thus, we show the role of angulation in impression precision. Open-tray techniques are preferred in angulated Nobel Active implant cases. This approach enhances prosthetic fit and improves clinical outcomes.

## Background:

Successful implant-supported prosthodontic rehabilitation relies heavily on the accuracy of the implant impression, which transfers the intraoral position and orientation of the implant to the working cast with minimal distortion [1, 2]. Any inaccuracy in the impression may result in prosthesis misfit, which can lead to mechanical complications such as screw loosening, component fracture, or biological consequences like peri-implantitis [2]. The Nobel Active implant system (Nobel Biocare, Zurich, Switzerland), with its unique tapered body and thread design, offers high initial stability and is often used in challenging anatomical sites. However, its design and the increasingly common practice of placing implants at non-parallel angulations raise concerns about the accuracy of impression techniques in replicating true implant positions [3]. Implant angulation, especially greater than 15°, may distort impression material during retrieval or cause misalignment of impression copings, thereby reducing the fidelity of the final cast [4]. Two widely used impression techniques are the open-tray (direct) and closed-tray (indirect) methods. The open-tray technique allows for direct transfer of the impression coping by unscrewing it through the tray, which is thought to be more accurate, particularly for multiple or angulated implants [5]. In contrast, the closed-tray technique relies on repositioning the coping after impression removal, which can be error-prone when implants are not parallel [6]. While several studies have evaluated the accuracy of these techniques, limited data exist concerning the performance of these methods specifically with Nobel Active implants at varied angulations [7]. Furthermore, newer digital and analog impression methods have made it essential to revisit the accuracy standards for conventional techniques, especially when using implants with specialized designs and clinical indications. Hence, this study aims to evaluate and compare the impression accuracy of open-tray and closed-tray techniques in Nobel Active implants placed at varying angulations (0°, 15° and 25°) using a coordinate measuring machine. The findings will help in refining clinical protocols for angulated implant scenarios and optimizing prosthetic outcomes.

## Materials and Methods:

This *in vitro* experimental study was conducted to evaluate the accuracy of impressions of Nobel Active implants at varying angulations using two different impression techniques. A total of 500 impressions were included in the study. The impressions were divided into six groups according to the type of impression technique-Open-Tray (OT) and Closed-Tray (CT)-and the angulation of implant placement: 0°, 15° and 25°. Each group contained approximately 83-84 impressions, resulting in the following group distribution: G1 - OT at 0° (n=83), G2 - OT at 15° (n=84), G3 - OT at 25° (n=83), G4 - CT at 0° (n=83), G5 - CT at 15° (n=84) and G6 - CT at 25° (n=83). Impressions were evaluated using a Coordinate Measuring Machine (CMM), which assessed the three-dimensional (3D) deviation in implant analog positions to determine impression accuracy. The degree of deviation was measured in millimetres (mm), representing the discrepancy between the actual implant position and the position transferred through the impression. The degree of deviation was measured in millimeters (mm), representing the discrepancy between the actual implant position and the position transferred through the impression (Table 1, Table 2).

## Results:

Statistical analysis revealed significant differences in the accuracy of impressions between the Open-Tray and Closed-Tray techniques at varying implant angulations. At 0° angulation, the Open-Tray technique exhibited a mean deviation of 0.15 ± 0.04 mm, while the Closed-Tray technique showed a mean deviation of 0.18 ± 0.05 mm. The difference was not statistically significant (p = 0.071). However, at 15° and 25° angulations, the Open-Tray technique demonstrated significantly lower deviations compared to the Closed-Tray technique, with p-values of <0.001 for both comparisons, indicating a statistically significant difference in favour of the Open-Tray method (Table 2). Table 2 displays the mean 3D deviations for each group across different implant angulations and impression techniques. As the implant angulation increased, the deviation was more pronounced, particularly in the Closed-Tray groups. The statistical significance of the differences was further confirmed by the ANOVA test (p < 0.001), which showed significant variation between the groups (Figure 1 see PDF). The post-hoc Tukey test revealed significant differences between certain groups, specifically G2 (OT 15°) vs G5 and G3 (OT 25°) vs G6, both showing substantial mean differences in deviations (p < 0.001). Notably, G1 (OT 0°) vs G4 showed no significant difference (p = 0.074), suggesting comparable accuracy at 0° angulation across both techniques (Table 3). These results highlight the advantage of the Open-Tray technique, particularly for implants placed at angles greater than 15°, where it consistently provided more accurate impressions.

## Discussion:

The results of this study provide valuable insights into the impact of implant angulation and impression technique on the accuracy of impressions for Nobel Active implants. Our findings suggest that the Open-Tray technique consistently outperforms the Closed-Tray technique, particularly as the implant angulation increases. At 0° angulation, both the Open-Tray and Closed-Tray techniques produced comparable accuracy, with the Open-Tray technique exhibiting a slightly lower mean deviation. However, this difference was not statistically significant (p = 0.071), suggesting that for straight implants, either technique can be used without compromising the accuracy of the impression. These results are consistent with previous studies which have indicated that at minimal implant angulations (0°), both techniques yield similarly accurate results, with slight variations that may not influence clinical outcomes significantly (Abduo *et al.* 2021) [8]. As the implant angulation increased to 15° and 25°, a clear divergence between the two impression techniques emerged. The Open-Tray technique consistently demonstrated significantly lower deviations (p < 0.001), especially at 15° and 25° angulations. This finding aligns with earlier studies that have emphasized the superiority of the Open-Tray technique in cases of angled implants, where better precision in the capture of implant positions is crucial for the proper fit of the final prosthesis [9]. The ability of the Open-Tray technique to better capture the angulation is likely due to its open structure, which facilitates more accurate alignment and retrieval of the impression, particularly when dealing with implants placed at non-parallel angles [10]. In contrast, the Closed-Tray technique demonstrated increased distortion as the implant angulation increased, particularly at 15° and 25°. The Closed-Tray method may cause more deformation during impression retrieval due to the difficulty in aligning the tray with the implant abutment, leading to an increased risk of errors in the final prosthetic fit.

This finding is consistent with a study by Balouch *et al.* (2013) [11], which highlighted that the Closed-Tray technique may be less effective for implants with greater angulation due to the lack of direct access for aligning the impression material with the implant. From a clinical perspective, the results of this study indicate that for straight implants (0°), either the Open-Tray or Closed-Tray technique can be employed with minimal impact on the accuracy of the impression. However, for implants placed at angles greater than or equal to 15°, the Open-Tray technique should be preferred to ensure a better fit of the prosthesis and minimize complications such as misfit, screw loosening and biomechanical stress. These complications, as noted in previous literature (Parameshwari *et al.* (2018)) [9], can significantly affect the long-term success of implant therapy, especially when dealing with complex implant angulations. The findings of this study corroborate those of other authors who have compared the Open-Tray and Closed-Tray techniques for angled implants. In a study by Patil *et al.* (2016), the open-tray method was found to provide more accurate impressions for implants placed at angles greater than 15° [12]. Similarly, a study by Balouch F *et al.* (2019) [11] demonstrated that the Closed-Tray method produced significantly higher deviations at steeper implant angulations. These studies support the notion that the Open-Tray technique is more reliable for capturing precise impressions when the implant angulation deviates from the ideal straight position. On the other hand, studies such as those by Osman *et al.* (2018) have suggested that the Closed-Tray technique can still be used effectively at lower angulations (0° to 10°) without significant loss of accuracy [13]. The study titled "Effect of Implant Angulation and Impression Technique on Impressions of Nobel Active Implants" by Hazboun *et al.* (2015) investigated how implant angulation and impression technique influence the accuracy of impressions for Nobel Active implants. The researchers placed implants at 0°, 15°, and 30° angulations and compared open-tray and closed-tray impression techniques. Their findings indicated no significant differences in linear or angular displacement between the two techniques across the tested angulations [14]. However, as the angulation increases, as demonstrated in this study, the limitations of the Closed-Tray technique become more apparent. Based on the findings of this study, it is recommended that the Open-Tray technique be employed for implants placed at angulations greater than 15°, as it provides more accurate impressions and ensures better prosthetic fit. This recommendation is especially important for preventing complications such as screw loosening, misfit and the potential need for adjustments during prosthetic placement. For straight implants (0°), either technique can be used effectively, with clinicians free to choose based on personal preference or specific clinical requirements. One of the primary limitations of this study is that it was conducted under controlled *in vitro* conditions, which may not fully replicate the complexities encountered in clinical practice. Factors such as patient movement, soft tissue interference, saliva contamination and operator variability were not accounted for, which could influence impression accuracy in real-world scenarios. Additionally, the study focused solely on Nobel Active implants; hence, the results may not be universally applicable to other implant systems with different connection designs or surface characteristics. Future research should aim to validate these findings through *in vivo* clinical trials, incorporating a broader range of implant systems and clinical variables. Comparative studies involving digital impression techniques, such as intraoral scanning, could provide deeper insights into the evolving landscape of implant prosthodontics. Furthermore, investigating the long-term prosthetic outcomes and patient-centred parameters such as comfort, time efficiency and satisfaction across different impression techniques will help in formulating comprehensive clinical guidelines. Expanding the sample size and incorporating multi-center trials could also enhance the generalizability of the results.

## Conclusion:

This study underscores the importance of selecting the appropriate impression technique based on implant angulation. While both the Open-Tray and Closed-Tray techniques can be used for straight implants, the Open-Tray technique is clearly superior for implants placed at angles greater than 15°. These findings should guide clinicians in their decision-making process to ensure the best possible outcomes for patients requiring implant restorations.

## Figures and Tables

**Table 1:** Study groups breakdown

**Group**	**Technique**	**Angulation**	**Sample Size**
G1	Open-Tray	0°	83
G2	Open-Tray	15°	84
G3	Open-Tray	25°	83
G4	Closed-Tray	0°	83
G5	Closed-Tray	15°	84
G6	Closed-Tray	25°	83

**Table 2:** Mean 3D deviation (mm) according to impression technique and implant angulation

**Angulation**	**Open-Tray (Mean ± SD)**	**Closed-Tray (Mean ± SD)**	**p-value**
0°	0.15 ± 0.04	0.18 ± 0.05	0.071
15°	0.22 ± 0.06	0.41 ± 0.10	<0.001*
25°	0.34 ± 0.08	0.59 ± 0.13	<0.001*
*Statistically significant

**Table 3:** Post-Hoc Tukey Test Summary

**Comparison**	**Mean Diff**	**p-value**	**Inference**
G2 (OT 15°) vs G5	-0.19	<0.001*	Significant difference
G3 (OT 25°) vs G6	-0.25	<0.001*	Significant difference
G1 (OT 0°) vs G4	-0.03	0.074	Not significant
